# Evaluation and clinical relevance of plan quality indices in intracavitary brachytherapy for cervical cancer

**DOI:** 10.12688/f1000research.166604.1

**Published:** 2025-09-09

**Authors:** Abhishek Krishna, Juvie Leona Lobo, Pooja MS, Bharat Sai Makkapati, Athiyamaan MS, Challapalli Srinivas, Sourjya Banerjee, Johan Sunny, Paul Simon, Dilson Lobo

**Affiliations:** 1 Department of Radiation Oncology, Kasturba Medical College Mangalore, Manipal Academy of Higher Education, Manipal, India

**Keywords:** Uterine Cervical Neoplasms; Brachytherapy; Radiotherapy, High-Energy; Radiotherapy Planning, Computer-Assisted; Radiation Dosage; Treatment Outcome; Radiation Injuries.

## Abstract

**Background:**

Cervical cancer remains to be a significant health concern globally, with India contributing to nearly one-quarter of cases worldwide. Intracavitary brachytherapy (ICBT) combined with external beam radiotherapy (EBRT) is the standard of care, optimizing tumour dose while sparing normal tissues. Plan quality indices such as Dose Homogeneity Index (DHI), Overdose Index (OI), Dose Non-uniformity Ratio (DNR), and Conformity Index (COIN) are crucial in evaluating treatment plans, yet their predictive clinical relevance remains underexplored.

**Methodology:**

In this prospective study, 84 patients with histologically confirmed cervical cancer undergoing definitive chemoradiation followed by CT-based HDR ICBT (Co-60) were evaluated. Plan indices were calculated per ICRU 89 and GEC-ESTRO guidelines. Correlation with tumour response (RECIST v1.1), toxicity (≥ grade 2 bladder/rectal), and clinical factors (FIGO stage, HRCTV volume, EQD2, BED, treatment duration) was assessed using t-tests, regression analyses, and Pearson’s correlation.

**Results:**

Mean DHI was 0.403; OI 0.358; DNR 0.594; COIN 0.636. Higher DHI (p=0.019) and lower OI (p=0.004) were associated with tumour response; DNR and COIN were not significant. No plan index predicted ≥ grade 2 bladder or rectal toxicity. COIN correlated poorly with other indices. Multiple regression showed no consistent predictors of dosimetric indices. Logistic regression did not establish any index as a definitive predictor of 3-month response.

**Conclusion:**

Plan quality indices provide useful quantitative insights into dose distribution in High dose rate intracavitary brachytherapy for cervical cancer. In this study, DHI and OI showed an association with early tumour response, while no significant correlation was observed between plan indices and treatment-related toxicity.

## Introduction

Cervical cancer continues to remain a significant public health concern globally, with an estimated 662,301 new cases and 348,874 deaths in 2022. ^1^ In countries like India, cervical cancer represents a major public health challenge. ^2^ One-quarter of all cervical cancer incidences globally have been identified in India. ^1^

Brachytherapy enhances local tumor control and overall survival while maintaining manageable toxicity levels. The combination of intracavitary brachytherapy (ICBT) and external beam radiotherapy (EBRT) constitutes the standard curative approach for cervical cancer. ^3^ This treatment approach offers a high therapeutic index by delivering a high dose to the tumour while sparing the nearby organs. ^4^

The global popularity of HDR brachytherapy with Co-60 is increasing. It utilizes a small Co-60 source that is similar in size to an Ir-192 source. ^5^ The success of ICBT depends on the precise and accurate delivery of brachytherapy to the target volume, which can be achieved through careful High Dose Rate (HDR) treatment planning. ^3^

Plan indices serve as important measures for evaluating the quality of radiation therapy plans and anticipating clinical outcomes. Among these, the dose-volume histogram (DVH) is frequently utilized, providing comprehensive insights into the dose distribution across both the target area and surrounding healthy tissues. ^6^ Additional indices include the Conformity Index (COIN), Dose Homogeneity Index (DHI), Dose Non-Homogeneity Ratio (DNHR), and Overall Index (OI). ^6– 8^ The COIN measures the conformity of the radiation dose distribution to the target volume, while the DHI and DNR assess the radiation dose homogeneity and steepness of dose gradients, respectively. ^7, 8^

The utilization of plan indices in radiation treatment planning offers a robust framework for objectively assessing the quality and efficacy of treatment plans. These indices, such as the dose-volume histogram (DVH), conformity index (COIN), dose homogeneity index (DHI), and others, provide quantitative metrics that evaluate various aspects of treatment plans, including precision, uniformity, and overall effectiveness. ^7^ By analysing the DVH, clinicians can ascertain whether the prescribed dose adequately covers the target while minimizing radiation exposure to critical structures, thus ensuring precision in treatment delivery.

Only a few studies in the literature have explored the plan indices and their significance in cervical cancer patients undergoing intracavitary brachytherapy. ^6– 8^ Despite the extensive research in cervical cancer treatment, there remains a lack of consensus regarding which plan indices are the most dependable and predictive for ICBT treatment planning. This uncertainty is partly due to conflicting results reported across numerous studies, which may arise from differences in the patient cohorts, treatment techniques, and evaluation criteria.

The objective of the research was to evaluate and ascertain the clinical significance of multiple plan indices in predicting treatment outcomes and toxicities for cervical cancer patients undergoing ICBT.

## Methodology

This was a prospective study conducted at a single tertiary care institution. The study enrolled patients with pathologically confirmed carcinoma of the cervix who were planned for definitive chemoradiation followed by intracavitary brachytherapy (ICBT). Institutional Ethics Committee approval was obtained before the start of the study, and all participants provided written informed consent. Eligible patients included those with histologically proven cervical cancer, deemed suitable for radical intent treatment. Exclusion criteria were metastatic disease, prior history of pelvic radiotherapy or chemotherapy, and inability to complete planned brachytherapy or follow-up assessments. Patients with incomplete medical records or inadequate imaging for plan evaluation were also excluded. All patients underwent baseline magnetic resonance imaging (MRI) for pelvic assessment before treatment initiation. External beam radiotherapy (EBRT) was delivered to the whole pelvis using 3DCRT or IMRT to a dose of 46 to 50 Gy in 23 to 25 fractions, with concurrent weekly cisplatin or carboplatin chemotherapy. In case a nodal boost was planned, an additional dose of 8 to 10 Gy in 4 to 5 fractions was delivered sequentially.

Upon completion of EBRT, patients were clinically assessed for brachytherapy suitability. Those selected for intracavitary brachytherapy (ICBT) underwent the procedure under spinal anesthesia using Fletcher-Suit applicators. A dose of 7 to 7.5 Gy per fraction × 3 fractions was prescribed.

Following applicator insertion, computed tomography (CT)-based simulation was performed. Delineation of high-risk clinical target volume (HRCTV), intermediate-risk clinical target volume (IRCTV), and organs at risk (OARs) was carried out according to GEC-ESTRO and IBS guidelines. ^3, 9^

### Treatment delivery

Intracavitary brachytherapy (ICBT) treatment was administered using Cobalt 60 high dose rate brachytherapy (HDRBT) system. The prescribed dosage adhered to the recomendations outlined in the International Commission on Radiation Units and Measurements (ICRU) 38 report. ^10^

Following completion of the planned treatment, patients were monitored through a structured follow-up schedule. Follow-up evaluations were done every 3 months for the first 2 years, every 6 months for the subsequent 3 years, and annually thereafter. Treatment response was assessed at 3 months post-therapy using clinical and radiological with an MRI. Response evaluation adhered to the criteria defined by RECIST version 1.1. ^11^ In cases where residual or suspicious lesions were detected, further confirmation was sought through biopsy and histopathological examination.

### Plan evaluation

Plan quality evaluation was performed using a set of standard dosimetric parameters derived from patients treatment plan. These parameters were used to calculate established brachytherapy plan indices as per ICRU 89 recommendations. ^12^

The following parameters were extracted for analysis: D90, defined as the dose received by the 90% of the target volume; V100%, representing the percentage of the total target volume receiving 100% of the total prescribed dose; V150%, the percentage of the target volume receiving at least 150% of the total prescribed dose; and V200%, the percentage of the target volume receiving at least 200% of the prescribed dose. ^12^Additionally, volumetric parameters included TV (total target volume), TVDref (target volume receiving 100% of the prescribed dose), TV1.5ref (target volume receiving 150% of the prescribed dose), and TV2ref (target volume receiving 200% of the prescribed dose). The Vref parameter represented the overall volume receiving 100% of the prescribed dose, inclusive of both the target and surrounding tissues. ^12^

The quality of dose distribution within the planned target volume was further evaluated as per ICRU 89 and GEC ESTRO recommendations using the Dose Homogeneity Index (DHI), Dose Non-Uniformity Ratio (DNR), Overdose Index (OI), and Conformity Index (COIN). ^9, 12^ The DHI was calculated as (TVDref − TV1.5ref ) divided by TVDref, providing a measure of the uniformity of dose distribution within the target volume; higher values indicated better homogeneity. The DNR was computed as the ratio of the target volume receiving ≥150% of the prescribed dose (TV1.5ref ) to the volume receiving 100% of the prescribed dose (TVref ), quantifying the extent of dose non-uniformity. The OI was defined as the ratio of the target volume receiving ≥200% of the prescribed dose (TV2ref ) to TVref, representing the extent of high-dose regions within the target. Finally, the COIN assessed both target coverage and dose conformity, calculated as the product of two factors: C1 (TVDref/TV), representing target coverage, and C2 (TVref/Vref ), reflecting dose conformity to the target volume.

### Statistical analysis

Descriptive statistics were used to summarize patient characteristics, treatment parameters, and dosimetric indices. Continuous variables were expressed as mean, standard deviation (SD), minimum, and maximum values. Categorical variables were presented as frequencies and percentages. Correlations among dosimetric indices (DHI, OI, DNR, and COIN) were assessed using Pearson’s correlation coefficient. To compare dosimetric indices between responders and non-responders, independent samples t-tests were performed. Differences in mean DHI, OI, DNR, and COIN between the two groups were analyzed, with statistical significance set at p < 0.05. Logistic regression analysis was conducted to evaluate whether dosimetric indices (DHI, OI, DNR, and COIN) could predict response at 3 months (complete vs progressive disease; complete vs partial response). Regression coefficients, standard errors, and p-values were reported. Ordinal regression analysis was performed to assess the association between dosimetric indices and the grade of rectal and bladder toxicity. Multiple linear regression was used to explore the relationship between each dosimetric index (DHI, OI, DNR, COIN) and potential clinical or treatment-related factors, including FIGO stage, pre-treatment tumor volume, HRCTV volume, BED to tumor, EQD2 to tumor, and overall treatment time. Regression estimates, standard errors, t-values, and p-values were reported for each predictor. Finally, logistic regression analysis was performed to assess the relationship between dosimetric indices and the incidence of grade >2 bladder and rectal toxicities. The association of each index with high-grade toxicity was evaluated, and statistical significance was considered at p < 0.05. All statistical analyses were conducted using SPSS (version 24). P-values < 0.05 were considered statistically significant in all cases.

## Results

A total of 84 patients with carcinoma of the cervix who underwent intracavitary brachytherapy were included in the study. The most common FIGO stage was IIB (45.2%), followed by IIIC1 (23.8%), IIIB (9.5%), IIA (6.0%), IVA (7.1%), IB2 (2.4%), IIIC2 (2.4%), IIA2 (1.2%), and IVA (2.4%). Histopathological evaluation showed squamous cell carcinoma in 77 patients (91.7%), adenocarcinoma in 5 patients (6.0%), and villoglandular variant in 2 patients (2.4%). Chemotherapy was administered concurrently in all patients, with 67 receiving cisplatin (79.8%) and 17 receiving carboplatin (20.2%). The average number of chemotherapy cycles was 4 (range 3–5) for carboplatin and 4 (range 1–6) for cisplatin ( Table 1).

**Table 1.** Patient characteristics.

Characteristics	N = 84	Percentage
**Age in years (Median)**	54	
**Stage**		
IB2	2	2.4%
IIA	5	6.0%
IIA2	1	1.2%
IIB	38	45.2%
IIIB	8	9.5%
IIIC1	20	23.8%
IIIC2	2	2.4%
IVA	2	2.4%
IVA	6	7.1%
**Chemotherapy**		
Carboplatin	17	20.2%
Cisplatin	67	79.8%
**Mean Chemotherapy Cycles**	4	
**Pathology**		
Adenocarcinoma	5	6.0%
Squamous Cell Carcinoma	77	91.7%
Villoglandular Carcinoma	2	2.4%
**Brachytherapy Dose Fractionation**	7.5Gy in 3 fractions	

The mean pre-treatment tumor volume was 61.66 cm ^3^ (SD = 60.94), with a minimum of 2.37 cm ^3^ and a maximum of 252.81 cm ^3^. The mean brachytherapy dose per fraction was 7.72 Gy (SD = 0.34), with a range from 7.00 Gy to 8.50 Gy. The calculated biologically effective dose (BED) to the tumor was 99.85 Gy (SD = 3.58), with an equivalent dose in 2 Gy fractions (EQD2) of 83.27 Gy (SD = 2.98). The EQD2 to the bladder, rectum, and sigmoid colon were 73.37 Gy (SD = 5.93), 73.48 Gy (SD = 2.76), and 58.90 Gy (SD = 4.71), respectively ( Table 2).

**Table 2.** Radiotherapy dose details.

	N	Mean	SD	Minimum	Maximum
**PRE TREATMENT TUMOUR VOLUME**	84	61.66	60.942	2.37	252.81
**BRACHY DOSE**	84	7.72	0.341	7.00	8.50
**EQD2 TUMOR (GY10)**	84	83.27	2.980	76.20	92.82
**BED TO TUMOR**	84	99.85	3.579	91.46	111.39
**EQD2 BLADDER (GY3)**	84	73.37	5.932	58.97	85.19
**EQD2 RECTUM (GY3)**	84	73.48	2.761	64.00	84.58
**EQD2 SIGMOID (GY3)**	84	58.90	4.707	51.20	70.41

**Figure 1.**
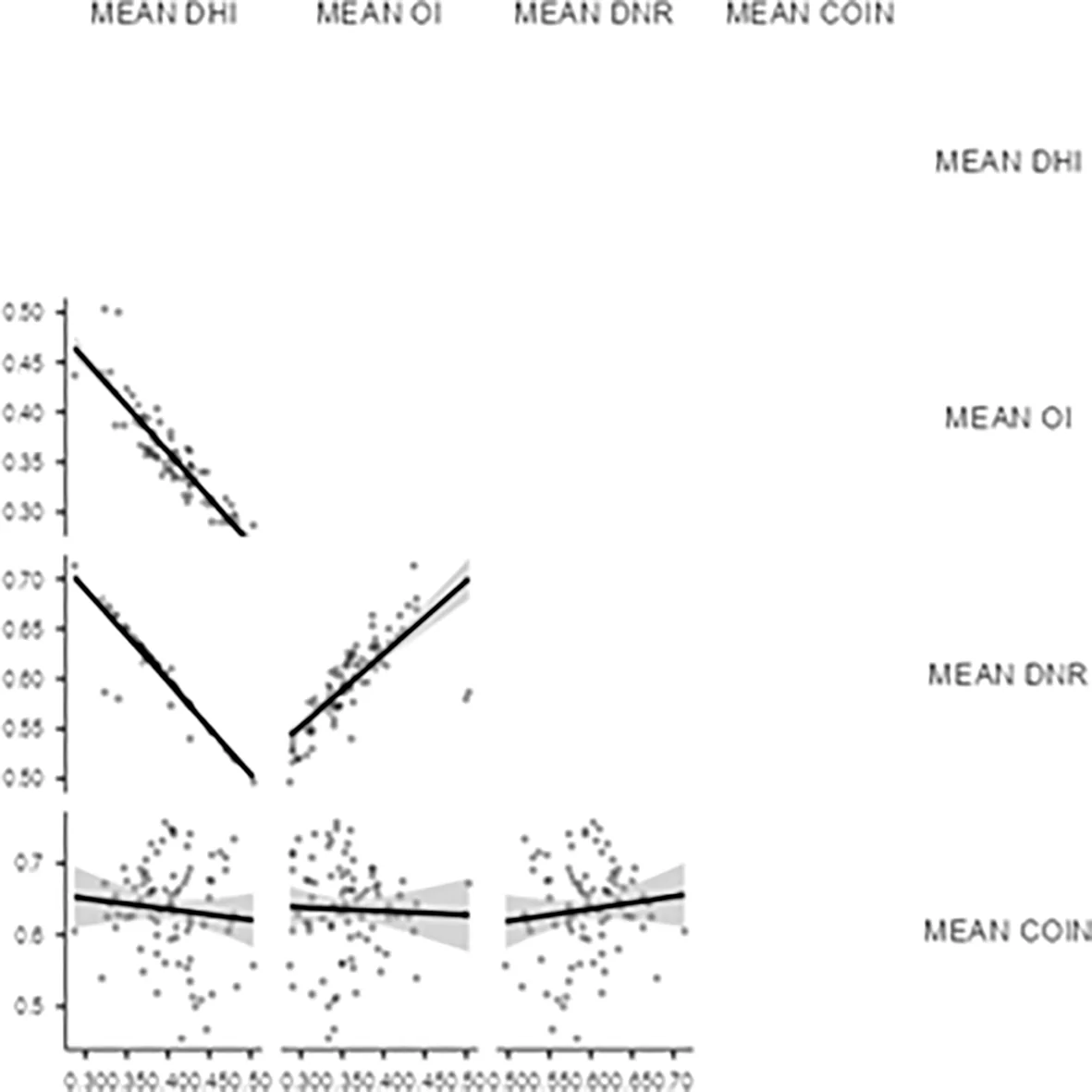
Correlation of various dosimetric indices.

Dosimetric indices for all patients were calculated. The mean dose homogeneity index (DHI) was 0.403 (SD = 0.043), mean overdose index (OI) was 0.358 (SD = 0.044), mean dose non-uniformity ratio (DNR) was 0.594 (SD = 0.042), and mean conformity index (COIN) was 0.636 (SD = 0.068) ( Table 3). Pearson’s correlation coefficient showed a statistically significant negative correlation between DHI and OI (r = -0.899, p < .001), and between DHI and DNR (r = -0.948, p < .001). A positive correlation was seen between OI and DNR (r = 0.761, p < .001). No statistically significant correlation was observed between COIN and the other indices ( Table 4 & Figure 1).

**Table 3.** Dosimetric indices details.

	N	Mean	SD	Minimum	Maximum
**MEAN DHI**	84	0.403	0.0433	0.287	0.503
**MEAN OI**	84	0.358	0.0443	0.287	0.503
**MEAN DNR**	84	0.594	0.0422	0.497	0.713
**MEAN COIN**	84	0.636	0.0684	0.455	0.757

**Table 4.** Correlation of various indices.

Variables	Pearson’s r	df	p-value	95% CI Lower	95% CI Upper
MEAN DHI vs MEAN OI	-0.899	82	< .001	-0.933	-0.847
MEAN DHI vs MEAN DNR	-0.948	82	< .001	-0.966	-0.921
MEAN DHI vs MEAN COIN	-0.094	82	0.393	-0.303	0.123
MEAN OI vs MEAN DNR	0.761	82	< .001	0.653	0.839
MEAN OI vs MEAN COIN	-0.034	82	0.758	-0.247	0.182
MEAN DNR vs MEAN COIN	0.106	82	0.337	-0.111	0.313

Independent samples t-test showed that DHI was significantly higher in responders compared to non-responders ( *p* = 0.019), while OI was significantly lower in responders ( *p* = 0.004). No significant differences were observed for DNR ( *p* = 0.085) and COIN ( *p* = 0.119) ( Table 5).

**Table 5.** Dosimetric parameters in responders and non responders.

Parameter	Response	Mean	SD	Statistic	df	p
**DHI**	Responders	0.406	0.0419	2.40	82.0	0.019
	Non Responders	0.367	0.0350			
**OI**	Responders	0.354	0.0414	-2.96	82.0	0.004
	Non Responders	0.403	0.0515			
**DNR**	Responders	0.592	0.0416	-1.74	82.0	0.085
	Non Responders	0.620	0.0311			
**COIN**	Responders	0.632	0.0695	-1.57	82.0	0.119
	Non Responders	0.674	0.0391			

Binary logistic regression for response at 3 months (complete vs progressive disease) was conducted. None of the indices reached statistical significance. Estimates were as follows: DHI = 1.723 (p = 0.978), OI = 27.535 (p = 0.403), DNR = 10.032 (p = 0.787), and COIN = 23.174 (p = 0.067) ( Table 6).

**Table 6.** Logistic regression.

Response at 3 months	Predictor	Estimate	SE	Z	p
**Complete Response - Progressive Disease**	**Intercept**	-34.843	60.75	-0.57351	0.566
	**MEAN DHI**	1.723	62.26	0.02768	0.978
	**MEAN OI**	27.535	32.92	0.83638	0.403
	**MEAN DNR**	10.032	37.08	0.27055	0.787
	**MEAN COIN**	23.174	12.66	1.83108	0.067
**Complete Response - Partial Response**	**Intercept**	0.750	135.32	0.00554	0.996
	**MEAN DHI**	-14.832	137.22	-0.10809	0.914
	**MEAN OI**	-28.980	33.57	-0.86325	0.388
	**MEAN DNR**	27.812	131.39	0.21168	0.832
	**MEAN COIN**	-6.243	7.37	-0.84743	0.397

Ordinal regression analysis for grade of rectal toxicity showed no statistically significant association for any of the indices: DHI (p = 0.502), OI (p = 0.891), DNR (p = 0.636), and COIN (p = 0.258). Similarly, there was no significant association for bladder toxicity: DHI (p = 0.104), OI (p = 0.221), DNR (p = 0.186), and COIN (p = 0.364) ( Table 7).

**Table 7.** Ordinal regression analysis for grade of rectal and bladder toxicity.

Rectal toxicity				
Predictor	Estimate	SE	Z	p
MEAN DHI	10.73	15.98	0.671	0.502
MEAN OI	-0.647	4.70	-0.138	0.891
MEAN DNR	7.72	16.33	0.473	0.636
MEAN COIN	-3.58	3.16	-1.132	0.258

Bladder toxicity				
Predictor	Estimate	SE	Z	p
MEAN DHI	18.6	11.4	1.62	0.104
MEAN OI	13.4	11.0	1.22	0.221
MEAN DNR	-7.09	5.36	-1.323	0.186
MEAN COIN	3.00	3.31	0.909	0.364

Correlation of dosimetric indices with FIGO stage, pre-treatment tumor volume, BED, EQD2, HRCTV volume, and overall treatment time was performed using multiple linear regression. For DHI, HRCTV volume showed a trend (p = 0.072), while no significant associations were seen for other variables. For OI, overall treatment time showed a borderline effect (p = 0.056). For DNR and COIN, no significant predictors were identified ( Table 8).

**Table 8.** Correlation of dosimetric indices with factors.

Parameter	Predictor	Estimate	SE	t	p
DHI	INTERCEPT	0.26084	0.12661	2.060	0.043
	STAGE	0.00757	0.00661	1.146	0.255
	BED TO TUMOR	0.00255	0.00810	0.315	0.754
	EQD2 TUMOR (GY10)	-0.00134	0.00981	-0.136	0.892
	HRCTV VOLUME	4.30e-4	2.36e-4	1.821	0.072
	PRE TREATMENT TUMOUR VOLUME	-9.74e−5	7.57e-5	-1.288	0.202
	OVERALL TREATMENT TIME IN DAYS	-7.04e−4	4.31e-4	-1.633	0.107
OI	INTERCEPT	0.47179	0.13202	3.574	<.001
	STAGE	-0.00691	0.00689	-1.003	0.319
	BED TO TUMOR	0.00278	0.00845	0.329	0.743
	EQD2 TUMOR (GY10)	-0.00507	0.01023	-0.495	0.622
	HRCTV VOLUME	-1.80e−4	2.46e-4	-0.730	0.468
	PRE TREATMENT TUMOUR VOLUME	1.14e-4	7.89e-5	1.451	0.151
	OVERALL TREATMENT TIME IN DAYS	8.71e-4	4.50e-4	1.938	0.056
DNR	INTERCEPT	0.66895	0.12534	5.3372	<.001
	STAGE	-0.00625	0.00654	-0.9563	0.342
	BED TO TUMOR	-0.00156	0.00802	-0.1938	0.847
	EQD2 TUMOR (GY10)	8.67e-4	0.00971	0.0892	0.929
	HRCTV VOLUME	-3.83e−4	2.34e-4	-1.6388	0.105
	PRE TREATMENT TUMOUR VOLUME	6.88e-5	7.49e-5	0.9190	0.361
	OVERALL TREATMENT TIME IN DAYS	7.45e-4	4.27e-4	1.7453	0.085
COIN	INTERCEPT	0.46598	0.2186	2.132	0.036
	STAGE	0.00621	0.0114	0.544	0.588
	BED TO TUMOR	0.00746	0.0140	0.533	0.595
	EQD2 TUMOR (GY10)	-0.00744	0.0169	-0.439	0.662
	HRCTV VOLUME	1.14e-4	4.08e-4	0.279	0.781
	PRE TREATMENT TUMOUR VOLUME	4.27e-5	1.31e-4	0.327	0.744
	OVERALL TREATMENT TIME IN DAYS	3.13e-4	7.44e-4	0.421	0.675

Toxicity analysis for grade >2 bladder and rectal events showed no statistically significant associations with any dosimetric index. For bladder: DHI (p = 0.482), OI (p = 0.624), DNR (p = 0.559), COIN (p = 0.400); and for rectum: DHI (p = 0.258), OI (p = 0.221), DNR (p = 0.460), COIN (p = 0.435) ( Table 9).

**Table 9.** Toxicity analysis.

Rectal toxicity > Grade 2				
Predictor	Estimate	SE	Z	p
Intercept	-94.53	88.83	-1.064	0.287
MEAN DHI	102.65	90.73	1.131	0.258
MEAN OI	33.77	27.61	1.223	0.221
MEAN DNR	60.76	82.32	0.738	0.460
MEAN COIN	4.30	5.51	0.780	0.435

Bladder toxicity > Grade 2				
Predictor	Estimate	SE	Z	p
Intercept	-61.55	84.09	-0.732	0.464
MEAN DHI	60.37	85.87	0.703	0.482
MEAN OI	13.07	26.68	0.490	0.624
MEAN DNR	45.65	78.14	0.584	0.559
MEAN COIN	5.01	5.96	0.842	0.400

## Discussion

The evolution of brachytherapy in cervical cancer has been influenced by the introduction of image-guided brachytherapy (IGBT), as advocated by the GEC-ESTRO working committee. ^9^ The GEC-ESTRO recommendations emphasize individualized dose optimization to achieve adequate target coverage while minimizing toxicity, which directly informs the interpretation of dosimetric indices such as DHI, DNR, OI, and COIN. The EMBRACE and EMBRACE II protocols have further standardized IGBT approaches, promoting consistent reporting of dose-volume parameters and encouraging adaptive planning based on anatomical changes during treatment. ^4^ The current study’s evaluation of plan quality indices within this framework aligns with the ongoing clinical emphasis on optimizing dosimetric quality and reinforcing the value of quantitative assessment in modern brachytherapy practice.

This study evaluated the dosimetric parameters and clinical utility of standard brachytherapy indices in patients undergoing high dose rate (HDR) intracavitary brachytherapy (ICBT) for cervical cancer. This comprehensive analysis highlights the associations between key indices such as Dose Homogeneity Index (DHI), Overdose Index (OI), Dose Non-Uniformity Ratio (DNR), and Conformity Index (COIN) with tumor response and toxicity outcomes. While brachytherapy remains a cornerstone in the curative management of locally advanced cervical cancer, the evolution of image-guided planning systems has made it possible to quantify dose distributions more precisely through these indices. Our findings offer valuable insights into the dosimetric quality of treatment plans and their potential impact on clinical outcomes. The mean values of the indices observed in our cohort — DHI (0.403), OI (0.358), DNR (0.594), and COIN (0.636) — suggest moderate dose uniformity and conformity in the overall treatment delivery. The moderate DHI and relatively elevated DNR and OI reflect the inherent limitations of standard tandem and ovoid-based ICBT in achieving highly homogeneous dose distributions. These findings are consistent with previous studies, which have shown that conventional intracavitary techniques, even with optimized planning, often result in significant dose gradients within the target volume due to applicator geometry and anatomical constraints. ^13, 14^

The COIN value of 0.636 reflects moderate conformity, which is a function of both adequate target coverage and avoidance of normal tissues. This index gains particular importance in the era of image-guided brachytherapy (IGBT), where the goal is not merely to deliver high doses to the high-risk clinical target volume (HR-CTV) but also to minimize spillover to adjacent organs at risk (OARs). The observed strong negative correlations between DHI and both OI (r = -0.899) and DNR (r = -0.948) are expected, as a more homogeneous dose distribution (high DHI) logically coincides with lower volumes receiving excessively high doses (low OI and DNR). The positive correlation between OI and DNR (r = 0.761) further supports this dose distribution trend, where plans with more overdose zones are likely to be less uniform. These findings are supported by studies in literature who have described similar relationships in interstitial and intracavitary HDR plans. ^15, 16^

Interestingly, COIN showed no statistically significant correlation with the other indices, highlighting its distinct role in plan evaluation. While DHI, OI, and DNR describe internal characteristics of dose distribution within the target, COIN encompasses spatial conformity — that is, the extent to which the high-dose volume coincides with the intended target and avoids non-target tissues.

The significant association of DHI and DNR with tumor response at 3 months — with lower DHI and higher DNR seen in patients with progressive disease — underscores the importance of achieving uniform dose coverage within the target. Previous studies have emphasized that non-uniform dose distributions with hotspots or cold spots may compromise treatment efficacy, either due to undertreated areas within the tumor or over-irradiated regions that lead to tissue necrosis and impaired tumor control. ^13, 15^

The trend toward statistical significance seen with COIN and OI in logistic regression models further supports their potential prognostic role. Although not reaching conventional levels of significance, these findings suggest that plans with poor conformity or higher overdose regions may influence tumor control, possibly through mechanisms such as uneven tumor sterilization or radiation-induced hypoxia in high-dose zones. The sample size of 84 patients, while adequate for exploratory analyses, may have limited the statistical power to detect small but clinically meaningful differences. These findings reinforce the need to integrate dosimetric indices into routine plan evaluation, not just as post-treatment analytics but as proactive tools to guide real-time decision-making during planning.

No statistically significant associations were observed between the evaluated dosimetric indices and grade ≥2 bladder or rectal toxicity in either ordinal or binary regression models. This may appear contrary to the intuitive expectation that indices reflecting high-dose volumes (e.g., OI and DNR) would correlate with increased toxicity. However, it is important to note that OAR doses were evaluated separately through standard D2cc measurements, which were within tolerance limits in most cases (mean EQD2 to bladder and rectum ~73 Gy). Additionally, previous studies by Kirchheiner et al. and Fokdal et al. have reported that D2cc values are more sensitive predictors of toxicity than global plan indices such as DNR or COIN. ^17, 18^ This suggests that while indices help in understanding dose distributions within the target, toxicity is more directly influenced by localized hot spots in the OARs. Nonetheless, the marginal trend seen for bladder toxicity with DHI (p = 0.104) in our data indicates a possible influence that warrants further investigation in larger cohorts.

Our regression models exploring predictors of the indices themselves (DHI, OI, DNR, COIN) did not identify any single parameter as a statistically significant determinant, although HR-CTV volume and treatment time showed near-significant trends for DHI and OI, respectively. This may suggest that anatomical and logistical treatment factors modestly influence dose distribution but are insufficient on their own to explain dosimetric variability. It also reflects the complexity of treatment planning in HDR brachytherapy, where multiple variables — applicator geometry, dwell times, optimization techniques — interact in nuanced ways.

The lack of correlation between FIGO stage and dosimetric indices is also notable, as it indicates that disease extent does not necessarily dictate the quality of dose distribution achievable with ICBT. This is likely because the applicator positioning and optimization methods are tailored to individual anatomy rather than disease stage per se.

The integration of dosimetric indices into brachytherapy planning represents a significant advancement in the quality assurance of radiotherapy. Indices such as DHI and COIN serve not only as measures of plan quality but also potentially as surrogates for predicting tumor control. Their objective, quantitative nature allows for plan comparisons across institutions and operators, aiding in standardization — a key challenge in HDR brachytherapy.

Moreover, the use of these indices aligns with recommendations from the GEC-ESTRO and ICRU 89 reports, which advocate for individualized, image-guided brachytherapy planning with thorough DVH-based evaluations. ^9, 12^ Studies such as EMBRACE and Retro EMBRACE have already demonstrated that consistent and optimized dosimetric parameters are associated with better local control and reduced toxicity, especially when EQD2 >85 Gy is achieved for HR-CTV without exceeding constraints for bladder and rectum. ^4, 18^

This study, while comprehensive, is limited by its retrospective design and single-center data. MRI-based brachytherapy planning, which is now considered standard in many centers ws used in only few patients in this study which may influence the generalizability of our results. Additionally, the small number of events in certain response and toxicity subgroups may reduce the statistical power of regression models. Future studies should focus on prospectively validating these findings in larger cohorts with MRI-guided planning, incorporating advanced tools such as automated dwell-time optimization and deformable image registration for cumulative dose assessment. Furthermore, integrating radiomics and machine learning approaches with dosimetric indices could enhance predictive modeling for response and toxicity.

## Conclusion

In conclusion, our study underscores the importance of incorporating brachytherapy indices such as DHI, OI, DNR, and COIN into intracavitary treatment planning for cervical cancer. These indices provide a nuanced understanding of dose distribution and may aid in refining treatment plans for better tumor response while minimizing toxicity. Continued emphasis on dosimetric quality assurance and individualized planning is essential to optimize outcomes in HDR brachytherapy.

## Consent

Written informed consent to take part in the research has been obtained from all patients when indicated. Consent for publication is not applicable as all data is anonymized, and no images are being published.

## Ethics statement

The Institutional Ethics Committee of Kasturba Medical College approved this study, Mangalore, vide Protocol No. IEC KMC MLR 12/2022/420). The study was conducted in accordance with the ethical guidelines and regulations set forth by the institution. The study has been conducted according to the principles expressed in the Declaration of Helsinki.

## Data Availability

FIGSHARE: https://doi.org/10.6084/m9.figshare.29302400.v1 Data are available under the terms of the Creative Commons Zero “No rights reserved” data waiver (CC0 4.0 Public domain dedication).
