# Supplementary material for: Evaluation and Clinical Relevance of Plan Quality Indices in Intracavitary Brachytherapy for Cervical Cancer
Source: F1000Res. 2026 Aug 19;14:882. Originally published 2025 Sep 9. [Version 2] doi: 10.12688/f1000research.166604.2 (PMC13560041; doi:10.12688/f1000research.166604.2)
Supplement: Peer review history

# Reviewer report: Reviewer response for version 2

**DOI:** 10.5256/f1000research.208287.r512727

**Published:** 2026-08-24

**Authors:** Sushma N

**Affiliations:** 1 Kidwai memorial Institute of Oncology Bangalore, Bangalore, India

no further comments to make

Is the work clearly and accurately presented and does it cite the current literature?

Yes

If applicable, is the statistical analysis and its interpretation appropriate?

Yes

Are all the source data underlying the results available to ensure full reproducibility?

Partly

Is the study design appropriate and is the work technically sound?

Partly

Are the conclusions drawn adequately supported by the results?

Yes

Are sufficient details of methods and analysis provided to allow replication by others?

Yes

Reviewer Expertise:

Radiation Physics

I confirm that I have read this submission and believe that I have an appropriate level of expertise to confirm that it is of an acceptable scientific standard.

# Reviewer report: Reviewer response for version 1

**DOI:** 10.5256/f1000research.183618.r506070

**Published:** 2026-08-04

**Authors:** Sushma N

**Affiliations:** 1 Kidwai memorial Institute of Oncology Bangalore, Bangalore, India

This study addresses an important and clinically relevant topic by evaluating the significance of  less commonly used plan quality indices in brachytherapy. The study is well designed, the methodology is adequately described, and the statistical analyses are generally appropriate. While the conclusions are supported by the presented data, a few minor revisions would further improve the clarity, reproducibility, and overall quality of the manuscript. My comments are as follows:

1. There appears to be an inconsistency in the study design. The Methods section describes the study as prospective, whereas the Discussion refers to it as retrospective. Please review and correct this to ensure consistency throughout the manuscript.
2. The limitations section could be expanded slightly. In addition to mentioning the single-center nature of the study and the limited use of MRI-based planning, it would be worthwhile to acknowledge the relatively short follow-up period (3-month response assessment) and the small number of toxicity events. These factors may have limited the ability to evaluate long-term clinical outcomes and detect meaningful associations with toxicity.
3. The manuscript would benefit from a careful language and proofreading review. There are a few minor grammatical and typographical errors that should be corrected before publication. For example, "recomendations" should be changed to "recommendations", the phrase "clinical and radiological with an MRI" could be reworded for clarity, and Table 1 appears to contain a duplicate entry for FIGO stage IVA.
4. The discussion could further elaborate on the clinical relevance of the findings. Although DHI and OI showed significant associations with early tumour response on univariate analysis, these associations were not maintained in the multivariable logistic regression. A brief discussion on how these indices may still be incorporated into routine plan evaluation, and whether future studies should aim to establish clinically useful threshold values, would strengthen the manuscript.

Is the work clearly and accurately presented and does it cite the current literature?

Yes

If applicable, is the statistical analysis and its interpretation appropriate?

Yes

Are all the source data underlying the results available to ensure full reproducibility?

Partly

Is the study design appropriate and is the work technically sound?

Partly

Are the conclusions drawn adequately supported by the results?

Yes

Are sufficient details of methods and analysis provided to allow replication by others?

Yes

Reviewer Expertise:

Radiation Physics

I confirm that I have read this submission and believe that I have an appropriate level of expertise to confirm that it is of an acceptable scientific standard.

# Response

**Published:** 2026-08-08

**Authors:** Dilson Lobo

**Affiliations:** Radiation Oncology, Kasturba Medical College Mangalore Manipal academy of Higher Education, Mangalore, Karnataka, India

**Response 1:**

 We confirm that the present study is a **retrospective study**.We have corrected this reviewed the manuscript to ensure that the study design is consistently described throughout. We will upload the revised manuscript.

**Response 2:**

  The limitations section has been expanded to acknowledge the relatively short follow-up period, with tumour response assessed at 3 months, as well as the small number of observed toxicity events. We have clarified that these factors limit our ability to draw conclusions regarding long-term clinical outcomes in the revised manuscript.

**Response 3:**

 We have revised the manuscript with respect to language and proof reading. Table 1 has also been reviewed, and the duplicate entry for FIGO stage IVA has been corrected. 

**Response 4:**

 The Discussion has been expanded to clarify the clinical relevance of our findings. Although DHI and OI demonstrated significant associations with early tumour response on univariate analysis, these associations were not retained after multivariable adjustment. Therefore, our findings should not be interpreted as establishing DHI or OI as independent predictors of tumour response.

# Reviewer report: Reviewer response for version 1

**DOI:** 10.5256/f1000research.183618.r449894

**Published:** 2026-01-30

**Authors:** Ehab Marouf Attalla

**Affiliations:** 1 Cairo University, Cairo, Egypt

Dear Editor

 The study evaluated Plan quality indices, such as Dose Homogeneity Index (DHI), Overdose Index (OI), Dose Non-uniformity Ratio (DNR), and Conformity Index (COIN), provide valuable insights into dose distribution in intracavitary brachytherapy (ICBT) for cervical cancer. Studies have shown that higher DHI and lower OI are associated with improved tumor response, while DNR and COIN are not significant predictors. Here are the advantages and disadvantages:

 Advantages:

  

- Useful quantitative insights: Plan quality indices provide valuable information about dose distribution in ICBT.
- Association with tumor response: DHI and OI showed significant association with early tumor response.
- Optimizing treatment plans: Indices can help optimize treatment plans to improve tumor control and reduce toxicity.

Disadvantages:

  

- Limited predictive value: No index was a definitive predictor of 3-month response or treatment-related toxicity.
- Poor correlation: COIN correlated poorly with other indices, limiting its usefulness.
- Need for further research: More studies are needed to establish the clinical relevance of plan quality indices.

The article highlights several research gaps in the field of cervical cancer treatment using intracavitary brachytherapy (ICBT):

1. Lack of consensus on plan indices: There's no agreement on which plan indices are most dependable and predictive for ICBT treatment planning.
2. Limited studies on plan indices: Few studies have explored plan indices and their significance in cervical cancer patients undergoing ICBT.
3. Conflicting results: Different studies report conflicting results, possibly due to differences in patient cohorts, treatment techniques, and evaluation criteria.
4. Limited understanding of dosimetric indices: The clinical significance of indices like DHI, OI, DNR, and COIN in predicting treatment outcomes and toxicities is not well understood.
5. Need for individualized planning: There's a need for personalized planning approaches incorporating dosimetric indices to optimize treatment outcomes.
6. Limited data on MRI-based brachytherapy planning: MRI-based brachytherapy planning is not widely used, and its impact on treatment outcomes is unclear.
7. Need for prospective validation: The study's findings need prospective validation in larger cohorts with MRI-guided planning.

Is the work clearly and accurately presented and does it cite the current literature?

Yes

If applicable, is the statistical analysis and its interpretation appropriate?

I cannot comment. A qualified statistician is required.

Are all the source data underlying the results available to ensure full reproducibility?

Yes

Is the study design appropriate and is the work technically sound?

Yes

Are the conclusions drawn adequately supported by the results?

Yes

Are sufficient details of methods and analysis provided to allow replication by others?

Yes

Reviewer Expertise:

Medical Physics

I confirm that I have read this submission and believe that I have an appropriate level of expertise to confirm that it is of an acceptable scientific standard.

## References

[1] : Research trends in cervical cancer brachytherapy: a bibliometric analysis. *Discover Oncology* .2025;16(1) : 10.1007/s12672-025-03287-4 10.1007/s12672-025-03287-4 40782269 PMC12335422

[2] : Combined Interstitial and Intracavitary High-Dose Rate Brachytherapy of Cervical Cancer. *Frontiers in Oncology* .2022;11: 10.3389/fonc.2021.809825 10.3389/fonc.2021.809825 PMC879386235096614
